# Urinary albumin excretion and prevalence of microalbuminuria in a general Chinese population: a cross-sectional study

**DOI:** 10.1186/1471-2369-15-165

**Published:** 2014-10-13

**Authors:** Liuxia Yan, Jixiang Ma, Xiaolei Guo, Junli Tang, Jiyu Zhang, Zilong Lu, Huicheng Wang, Xiaoning Cai, Linhong Wang

**Affiliations:** National Center for Chronic and Noncommunicable Disease Control and Prevention, Chinese Center for Disease Control and Prevention, Beijing, 100050 China; Academy of Preventive Medicine, Shandong University, Jinan, 250014 China; Shandong Center for Disease Control and Prevention, Jinan, 250014 China; Chinese Center for Disease Control and Prevention, Beijing, 102206 China

**Keywords:** Diabetes, Hypertension, Microalbuminuria

## Abstract

**Background:**

Microalbuminuria has been shown to be a risk factor for cardiovascular and renal disease in patients with hypertension and diabetes as well as in the general population. Urinary albumin excretion over 24 h is considered a ‘gold standard’ to detect microalbuminuria. Few studies have used 24-h urinary albumin excretion to analyze the prevalence of and related factors for microalbuminuira in a general Chinese population.

**Methods:**

This study included 1980 adults aged 18–69 years from the Shandong-Ministry of Health Action on Salt and Hypertension (SMASH) Project 2011 survey. Blood pressure, height, weight and waist circumference were measured, and a venous blood and timed 24-h urine samples were collected from each participant. Linear and logistic regression analyses were used to test associations between established cardiovascular risk factors and microalbuminuria.

**Results:**

The median (25th–75th percentile) of 24-h urinary albumin excretion was 6.1 mg/d (4.5–8.7 mg/d) for all adults, 6.0 mg/d (4.4–8.5 mg/d) for men and 6.2 mg/d (4.6–8.9 mg/d) for women. The overall prevalence of microalbuminuria was 4.1% (95% confidence interval [CI]: 3.2–5.0%), 3.7% (95% CI: 2.9–4.5%) for men and 4.6% (95% CI: 3.7–5.5%) for women. Microalbuminuria was present in 8.1% (95% CI: 6.9–9.3%) of individuals with hypertension, 11.4% (95% CI: 10.0–12.8%) of those with diabetes and 15.6% (95% CI: 14.0–17.2%) of those with both. Multiple logistic regression analysis indicated that systolic blood pressure (odds ratio [OR] 1.02; 95% CI: 1.01–1.03) and fasting blood glucose (OR 1.19; 95% CI: 1.05–1.35) were the independent risk factors for microalbuminuria.

**Conclusions:**

Adults in the general population of Shandong Province have a moderate prevalence of microalbuminuria. Those with hypertension and diabetes are at high risk of having microalbuminuria, suggesting the need for screening and early intervention for microalbuminuria among these individuals.

## Background

Microalbuminuria (MAU), an abnormal increase in the urinary excretion of albumin, is a risk factor for cardiovascular and renal disease in patients with hypertension and diabetes [1–4]. Early intervention for MAU among the patients with hypertension and diabetes has been shown to reduce their risks of progression of renal and cardiovascular complications [5, 6]. International guidelines therefore recommend that patients with hypertension and diabetes be screened for MAU, enhancing the benefits of early diagnosis and treatment [7, 8].

MAU has also been shown to be predictive of cardiovascular events and all cause mortality in the general population [9–11]. In middle-aged to elderly-aged Chinese adults, the risk of cardiovascular mortality is about three-fold higher in individuals with than without MAU [9]. Additionally, MAU was independently associated with other cardiovascular risk factors [12, 13]. Screening for MAU may result in the earlier detection and treatment of undiagnosed cardiovascular disease [13, 14].

The amount of urinary albumin excreted over a 24-h period is considered the ‘gold standard’ for defining MAU [3]. However, owing to the inconvenience and cumbersomeness of 24-h urine collection, spot urine measurements of albumin concentration or albumin to creatinine ratio are used as alternatives in clinical practice [15–17]. Indeed, the previous studies of MAU in Chinese adults were assessed using the alternative methods, not by measuring 24-h urinary albumin excretion. This study therefore assessed the prevalence of MAU in a general Chinese population by analyzing 24-h urine secretion, as well as analyzing the association between MAU and established risk factors for cardiovascular disease.

## Methods

### Subjects

The study subjects had participated in the Shandong-Ministry of Health Action on Salt and Hypertension (SMASH) project 2011 survey; the methods used for this survey have been described in detail [18]. Using a stratified multi-stage cluster sampling method, 2112 adults aged 18–69 years were selected from 20 counties and districts across Shandong Province. All the participants were required to complete a face-to-face questionnaire survey, undergo an anthropometric examination and provide fasting blood and timed 24-h urine samples. This study was conducted according to the guidelines of the Declaration of Helsinki, and all procedures involving human subjects were approved by the Ethics Committee of the Shandong Center for Disease Control and Prevention. Written informed consent was obtained from each subject.

### Anthropometric measurements

Physical examinations, including measurements of height, weight, waist circumference and blood pressure, were performed by trained health professionals. Weight was measured barefoot and in light-clothing. Body mass index (BMI) was calculated as weight in kilograms divided by the square of height in meters. Underweight, normal weight, overweight and obesity were defined as BMI < 18.5 kg/m^2^, ≥18.5 but <24 kg/m^2^, ≥24 but <28 kg/m^2^ and ≥28 kg/m^2^ respectively according to Chinese guidelines [19].

Blood pressure was measured in a sitting position three times every 5 min on one occasion using an electronic sphygmomanometer (HEM-7071, Omron Corporation, Japan), with the average of the three measures defined as individual blood pressure. Hypertension was defined as systolic blood pressure (SBP) ≥140 mmHg or diastolic blood pressure (DBP) ≥90 mmHg, or self-reported taking of anti-hypertensive medications [20].

### Blood sample collection and biochemical assays

A morning sample of venous blood was drawn from each participant and centrifuged within 2 h of collection. Participants with fasting blood glucose ≥ 6.1 mmol/L were invited to return for an oral glucose tolerance test (OGTT) on another day, at which time 2 h postload blood glucose (2hPBG) was tested.

Serum samples were frozen at -80°C. All blood and 24-h urine sample were assessed by ADICON Clinical Laboratory Inc., Jinan, Shandong. Serum glucose, total cholesterol, high-density lipoproteins, low-density lipoproteins and triglycerides were measured by standard laboratory methods on an Olympus AU640.

Diabetes was diagnosed according to the standard of the American Diabetes Association (2003) [21]. Participants were defined as having type 2 diabetes if they had fasting serum glucose ≥7.0 mmol/L, 2hPBG ≥11.1 mmol/L, or validated history of diabetes as diagnosed by a physician. Dyslipidaemia was defined as elevated serum concentrations of total cholesterol (TC) ≥6.1 mmol/L, and/or triacylglycerol (TG) ≥ 2.26 mmol/L, and/or LDL-cholesterol (LDL-C) ≥4.14 mmol/L, and/or a decreased HDL-cholesterol (HDL-C) concentration of <1.0 mmol/L as described by the American National Cholesterol Education Program (Adult Treatment Panel III) [22].

### 24-h urine collection and measurements

Participants were instructed on the methods of collection of a standard 24-hour urine sample. Each participant was given a standard plastic container containing about 1 g boric acid as a preservative. Participants were instructed to discard the first void and collect all the urine during the following 24 h in the container. A local health professional recorded the starting and ending times of each collection and determined the exact duration of collection. Each participant was interviewed using a standard questionnaire to assess the completeness of urine collection. Urine volume was measured on a standard platform at each field site by a laboratory technician. The collected urine samples were kept in a freezer at -20°C and were delivered to ADICON Clinical Laboratory Inc for laboratory testing. Individual urinary albumin and creatinine excretion were calculated as the products of their concentrations in the urine and urinary volume, corrected to 24 h.

### Evaluation of albuminuria

Urinary creatinine excretion was assessed using the picric acid method, and 24-h urinary albumin excretion (UAE) was assessed using an immunonephelometric method, both on an Olympus AU640 Analyzer. The albumin to creatinine ratio (ACR) was calculated, as were the agreements between albuminuria as determined by UAE and by ACR. For UAE, <30 mg/d, 30–299 mg/d and ≥300 mg/d were defined as normal, microalbuminuria and macroalbuminuria. The corresponding value for ACR was <30 mg/g, 30–299 mg/g and ≥300 mg/g, respectively.

Each 24-h urine sample was assessed for completeness using urinary volume and gender specific urinary creatinine cut-off point. Incompleteness was defined as 24-h urinary volume < 500 ml, and/or 24-h urinary creatinine < 1.91 or >18.27 mmol in men, or < 1.36 or >14.28 mmol in women, with these samples excluded from analysis.

Of the 2112 participants, 88 provided incomplete 24-h urine collection, and 44 failed to provide the blood sample. Thus, the study involved 1980 participants.

### Statistical analysis

Normally distributed and continuous variables were expressed as mean ± standard deviation (SD), and the differences between gender were assessed by t test. Mean and percentile values (i.e. P5, P25, P50, P75 and P95) of 24-h UAE were analyzed. The prevalence of microalbuminuria was reported as a percentage with 95% confidence intervals (CI). Of the 1980 participants, only two were classified as having macroalbuminuria, therefore, only factors associated with the prevalence of microalbuminuria were analyzed with differences between the proportions of subjects with microalbuminuria assessed by Fisher’s Exact test or the Chi-square test. Agreement in the classification of albuminuria by measurements of UAE and ACR was summarized by Cohen’s k [23].

Two methods were used for multiple regression analysis. First, a stepwise multiple linear regression analysis was performed. Because UAE was not normally distributed, log(n)UAE was considered the dependent variable, with age, gender, smoking (yes/no), BMI, SBP(mmHg), FBG(mmol/l) and TC(mmol/l) considered independent variables. The regression model also analyzed HDL-C, LDL-C and TG concentrations as covariates. Second, multiple stepwise logistic regression analysis was performed to assess factors associated with microalbuminuria. The dependent variable was microalbuminuria (yes/no), with the covariates the same as those described above.

Statistical analyses were performed with SAS 9.3 (SAS Institute Inc., Cary, North Carolina, USA). A P value < 0.05 was considered statistically significant.

## Results

### Clinical characteristics of the study population

Out of the 1980 participants, 52.4% were male, and their average age was 41 years old (SD = 14.0 years). Approximately 25% of the participants were hypertensive, with a median (Q1–Q3) hypertension duration of 3.0 (1.3–7.5) years. About 6.2% had diabetes, and 23.6% had dyslipidaemia. The average BMI was 24.5 kg/m^2^ and 17.8% of the participants were obese (Table 1).

**Table 1:** **Characteristics of the study population by gender**

	Men (n 1038)	Women (n 942)	Total (n 1980)	P
Age (years)	41.4 ± 14.2	41.4 ± 13.6	41.4 ± 14.0	0.76
Current smoking,%	47.4	2.7	26.1	<0.001
BMI (kg/m^2^)	24.5 ± 3.9	24.5 ± 3.9	24.5 ± 3.9	0.94
Waist circumference (cm)	85.5 ± 11.4	81.6 ± 10.8	83.7 ± 11.3	<0.001
Blood pressure				
SBP (mmHg)	124.7 ± 17.9	118 ± 19.2	121.5 ± 18.8	<0.0001
DBP (mmHg)	80.5 ± 11.5	77.1 ± 11.2	78.9 ± 11.5	<0.0001
Serum FBG (mmol)	5.5 ± 1.2	5.5 ± 1.1	5.5 ± 1.1	0.50
Dyslipidemia,%	28.0	18.8	23.6	<0.0001
TC (mmol)	4.4 ± 0.9	4.4 ± 1.0	4.4 ± 0.9	0.74
High TC,%	3.8	4.1	3.9	0.60
HDLC (mmol)	1.4 ± 0.4	1.5 ± 0.3	1.4 ± 0.3	0.10
Low HDL,%	14.6	8.3	11.3	<0.0001
LDLC (mmol)	2.2 ± 0.6	2.2 ± 0.6	2.2 ± 0.6	0.30
High LDL,%	0.6	1.0	0.8	0.33
TG (mmol)	1.6 ± 1.9	1.2 ± 1.1	1.4 ± 1.6	<0.001
High TG,%	15.8	10.7	13.4	0.0008
Hypertension,%	25.0	22.2	23.7	0.15
Diabetes,%	5.8	6.7	6.2	0.40
Obese,%	17.6	18.0	17.8	0.81
Urinary excretion				
Volume (ml/d)	1582 ± 660	1501 ± 587	1544 ± 627	0.004
Creatinine (mmol/d)	9.8 ± 3.3	7.5 ± 2.2	8.7 ± 3.1	<0.001

### Distribution of 24-h UAE

The overall median (P25-P75) 24-h UAE was 6.1 (4.5–8.7) mg/d, 6.0 (4.4–8.5) mg/d for men and 6.2 (4.6–8.9) mg/d for women. The median (P25-P75) 24-h UAEs among subjects with hypertension and diabetes were 6.7 (4.8–10.8) mg/d and 7.6 (5.3–11.3) mg/d respectively (Table 2).

**Table 2:** **24-h urinary albumin excretion (mg/d) and prevalence of microalbuminuria (%, 95% CI) by selected risk factors**

	24-h urinary albumin excretion (mg/d)							Microalbuminuria	P value
	N	Mean	P5	P25	P50	P75	P95	% (95% CI)	
Total	1980	10.5	2.9	4.5	6.1	8.7	24.9	4.1 (3.2-5.0)	-
Gender									
Men	1038	10.4	2.9	4.4	6.0	8.5	24.6	3.7 (2.9-4.5)	0.32
Women	942	10.7	3.0	4.6	6.2	8.9	26.2	4.6 (3.7-5.5)	
Age (years)									
18 ~ 29	499	8.7	2.4	4.3	5.7	8.3	22.9	3.0 (2.2-3.8)	0.49
30 ~ 39	500	10.1	3.2	4.7	6.1	8.9	24.3	4.6 (3.7-5.5)	
40 ~ 49	385	11.8	3.0	4.6	6.1	8.8	26.7	3.9 (3.0-4.8)	
≥50	596	11.5	2.9	4.5	6.3	8.9	28.7	4.7 (3.8-5.6)	
BMI									
Low weight	70	9.0	2.4	4.1	5.1	7.2	13.3	1.4 (0.9-1.9)	0.10
normal	885	9.7	2.8	4.4	6.0	8.4	21.0	3.5 (2.7-4.3)	
overweight	670	10.2	3.0	4.4	6.1	8.8	25.1	3.9 (3.0-4.8)	
obese	353	13.4	3.1	5.0	6.7	10.1	53.3	6.2 (5.1-7.3)	
Current smoker									
Yes	517	10.4	2.8	4.2	5.7	8.2	24.2	3.5 (2.7-4.3)	0.42
No	1463	10.6	3.0	4.6	6.2	8.9	25.1	4.3 (3.4-5.2)	
Hypertension									
Yes	468	15.5	3.2	4.8	6.7	10.8	50.2	8.1 (6.9-9.3)	<0.0001
No	1512	9.0	2.8	4.4	6.0	8.2	19.4	2.8 (2.1-3.5)	
Diabetes,%									
Yes	123	18.1	3.2	5.3	7.6	11.3	68.2	11.4 (10.0-12.8)	<0.0001
No	1857	10.0	2.9	4.5	6.0	8.6	22.9	3.6 (2.8-4.4)	
High TC,%									
Yes	78	10.6	3.2	5.0	6.5	9.4	40.8	7.7 (6.5-8.9)	0.10
No	1902	10.5	2.9	4.5	6.0	8.7	24.6	3.9 (3.0-4.8)	
High LDL,%									
Yes	15	8.6	2.1	3.8	7.9	9.7	30.0	6.7 (5.6-7.8)	0.62
No	1965	10.5	2.8	4.5	6.1	8.7	24.8	4.1 (3.2-5.0)	
Low HDL,%									
Yes	224	13.3	2.8	4.6	6.1	9.6	26.9	4.9 (3.9-5.9)	0.51
No	1756	10.1	2.9	4.5	6.1	8.7	24.8	4 .0(3.1-4.9)	
High TG,%									
Yes	265	12.3	3.1	4.8	6.4	10.0	35.0	6.0 (5.0-7.0)	0.10
No	1715	10.2	2.8	4.5	6.0	8.6	23.5	3.8 (3.0-4.6)	

### Prevalence of microalbuminuria

The prevalence of MAU among general Shandong adults was 4.1% (95% CI: 3.2–5.0%), 3.7% (95% CI: 2.9–4.5%) for men and 4.6% (95% CI: 3.7–5.5%) for women. MAU was present in 8.1% (95% CI: 6.9–9.3%) of the participants with hypertension, and in 11.4% (95% CI: 10.0–12.8%) of those with diabetes, with the prevalence in each of these subgroups significantly higher than in subgroups without hypertension or diabetes, respectively (P < 0.0001) (Table 2). The prevalence of MAU in participants with both hypertension and diabetes was 15.6% (95% CI: 14.0–17.2%).

**Figure 1:**
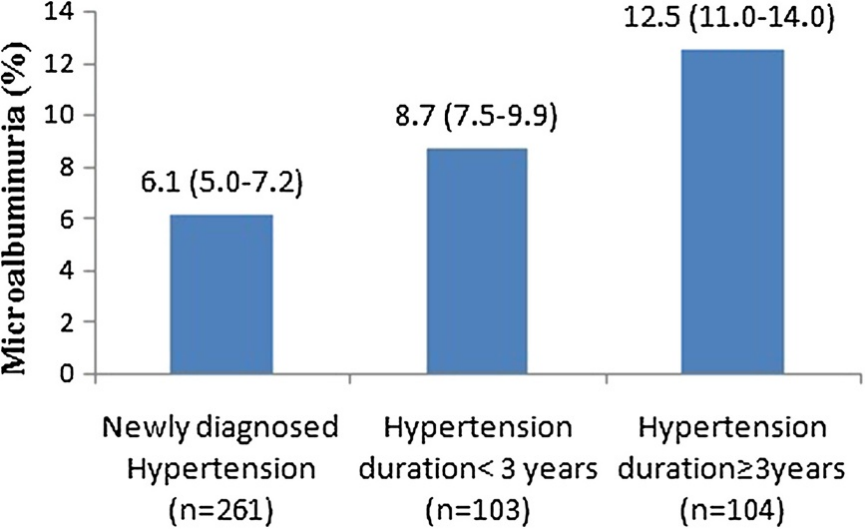
**Unadjusted prevalence of microalbuminuria (%, 95% CI) by duration of hypertension.**

The prevalence of MAU increased with the duration of hypertension (P_trend_ = 0.03). Relative to median disease duration (3 years), the prevalence of MAU was 8.7% (95% CI:7.5–9.9%) in subjects with hypertension <3 years and 12.5% (95% CI:11.0–14.0%) in subjects with hypertension >3 years (Figure 1).

**Figure 2:**
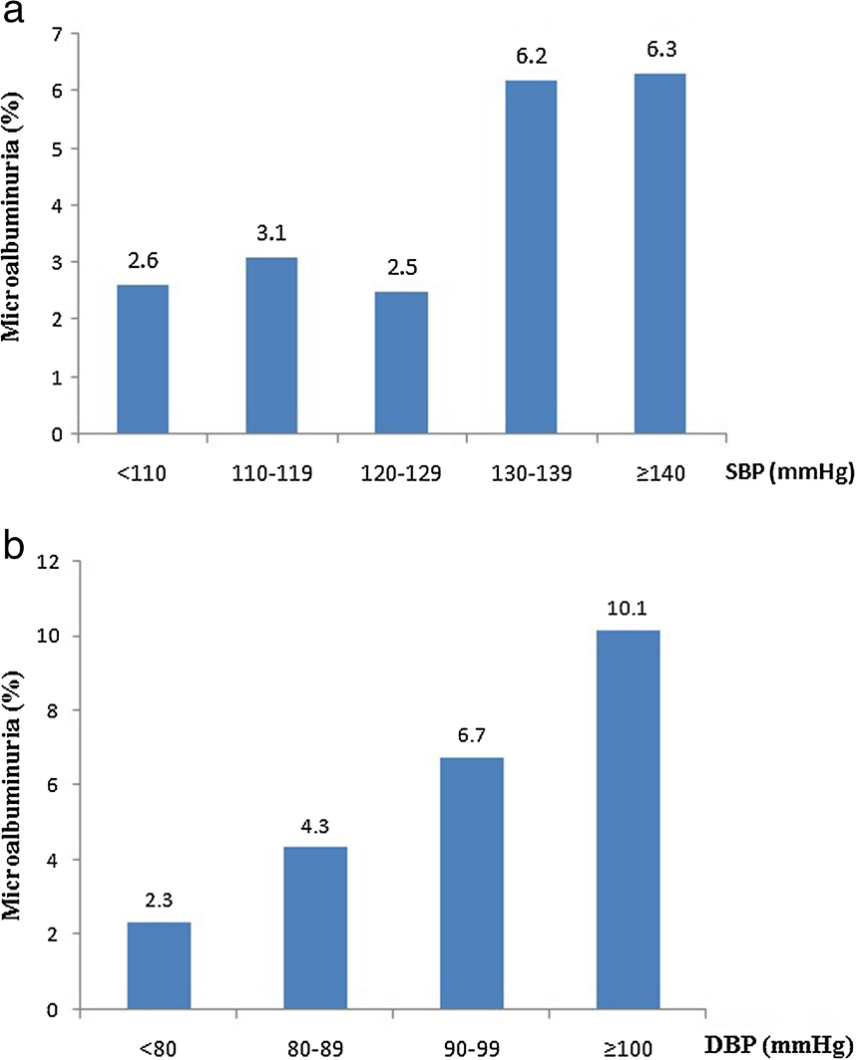
**Unadjusted prevalence of microalbuminuria (%) by SBP and DBP levels in the participants not treated with anti-hypertensive drugs. a**. Unadjusted prevalence of microalbuminuria by SBP level (<110 mmHg, 110-119 mmHg, 120-129 mmHg, 130–139 mmHg, ≥140 mmHg) in participants not treated with anti-hypertensive drugs. The rates of MAU (%, 95% CI) in these groups were 2.6% (95% CI: 1.9–3.3%), 3.1% (95% CI: 2.3–3.9%), 2.5% (95% CI: 1.8–3.2%), 6.2% (95% CI: 5.1–7.3%), 6.3% (95% CI: 5.2–7.4%). **b**. Unadjusted prevalence of microalbuminuria by DBP level (DBP < 80 mmHg, 80-89 mmHg, 90-99 mmHg, ≥100 mmHg) in the participants not treated with anti-hypertensive drugs. The rates (%, 95% CI) of MAU in these groups were 2.3% (95% CI: 1.6–3.0%), 4.3% (95% CI: 3.4–5.2%), 6.7% (95% CI: 5.6–7.8%), 10.1% (95% CI: 8.8–11.4%).

Among the participants not receiving anti-hypertensive treatment, there were linear trend relationships between the prevalence of MAU and SBP (P_trend_ = 0.003) and DBP (P_trend_ < 0.0001) levels (Figure 2).

**Figure 3:**
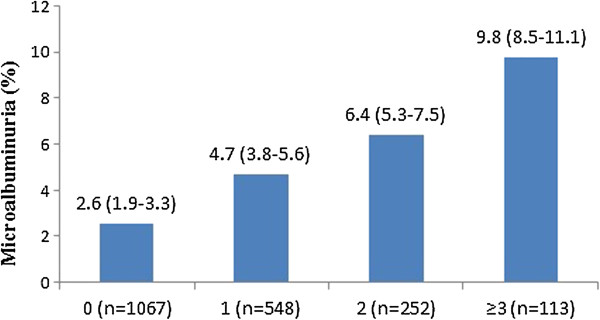
**Unadjusted prevalence of microalbuminuria (%, 95% CI) by number of cardiovascular disease risk factors, including hypertension, diabetes, dyslipidaemia and obesity.**

The prevalence of MAU increased with numbers of cardiovascular risk factors (P_trend_ < 0.001). Its prevalence among the participants without hypertension, diabetes, obesity or dyslipdaemia was 2.6% (95% CI: 1.9–3.3%), whereas its prevalence in the participants with >3 risk factors was 9.8% (95% CI: 8.5–11.1%) (Figure 3).

### Agreement between albuminuria assessed by measuring of UAE and ACR

Table 3 shows the agreement between the prevalence of albuminuria as determined by measuring of UAE and ACR. The overall k index was 0.86 (95% CI: 0.80–0.91).

**Table 3:** **Agreement of albuminuria defined by 24 h urinary albumin excretion and 24 h albumin to creatinine ratio**

Albumin to creatinine ratio (ACR)		Urinary albumin excretion (UAE)						(95% CI)
		Normal (<30 mg/24 h)		Microalbuminuria (30-299 mg/24 h)		Macroalbuminuria (≥300 mg/24 h)		
		No.	%	No.	%	No.	%	
Normal	<30 mg/g	1882	99.2	7	8.6	0	0	0.86 (0.80-0.91)
Microalbuminuria	30-299 mg/g	15	0.8	72	88.9	0	0	
Macroalbuminuria	≥300 mg/g	0	0	2	2.5	2	100.0	

### Risk factors for 24-h UAE and microalbuminuria

Linear regression analysis indicated that smoking, BMI, SBP and LDL-C were independently associated with the log(n)UAE. BMI and SBP were positively associated with UAE, while smoking and LDL-C were negatively associated (Table 4).

**Table 4:** Linear regression analysis of 24 h urinary albumin excretion with related covariates*

Covariates	β	P value
Smoking (yes/no)	-0.0008	0.02
BMI (kg/m^2^)	0.0165	0.0001
SBP (mmHg)	0.0043	<0.0001
LDL-C (mmol/l)	-0.0814	0.002

*: log(n) UAE was used as the dependent variable in the linear regression model, with the stepwise method used for selection of variables. The selected variables included age, gender, smoking (yes/no), BMI, SBP (mmHg), FBG (mmol/l), TC (mmol/l), HDL-C (mmol/l), LDL-C (mmol/l) and TG (mmol/l).

Multiple logistic regression analysis showed that SBP and FBG were independent risk factors for the development of MAU, FBG was the most important risk factor for MAU progression, with an odds ratio [OR] of 1.19 (95% CI: 1.05–1.35) (Table 5).

**Table 5:** Multiple logistic regression analysis of microalbuminuria with related covariates*

Covariates	OR (95% CI)	P value
SBP (mmHg)	1.02 (1.01-1.03)	<0.001
FBG (mmol/L)	1.19 (1.05-1.35)	0.006

*: Stepwise method was used for the variable selection. The selected variables included age, gender, smoking (yes/no), BMI, SBP (mmHg), FBG (mmol/l), TC (mmol/l), HDL-C (mmol/l), LDL-C (mmol/l) and TG (mmol/l).

## Discussion

Albuminuira, a component of the increasing disease burden of chronic kidney disease in China, has been a public health concern. This survey of community-dwelling Chinese adults aged 18 to 69 years and living in Shandong Province found that 4.1% had MAU, as defined by 24-h UAE, with MAU being more prevalent in the subjects with hypertension (8.1%) and diabetes (11.4%). Disease duration, blood pressure and serum glucose level were associated with MAU; therefore, both blood pressure and glucose should be controlled as much as possible to prevent the progression of MAU.

The prevalence of MAU in Shandong adults was lower in other regions in China (4.5–15%) [12, 24–26]. This discrepancy may be associated with geographic regional differences or the characteristics of the sampled population (e.g. age and cardiovascular disease profiles). In addition, MAU was defined in our study by 24-h UAE, whereas in other studies, MAU was defined by ACR or UAC. These measurements were moderately correlated and were comparable in predicting for cardiovascular disease mortality in prospective studies [17]. We observed substantial agreement of MAU classifications by measuring UAE and ACR in the same timed 24-h urine samples. However, there were still systematic differences in the classification of albuminuria prevalence [27]. Validation studies are required to determine the correlation between MAU as determined by 24-h UAE, as determined by ACR or UAC from untimed spot urine samples in Chinese population.

In agreement with previous studies, we found that hypertensive individuals were at high risk for MAU, with blood pressure linearly associated with MAU [12, 24, 28, 29]. A recent national hypertension survey in China found that blood pressure was controlled under 140/90 mmHg in 9.3% of hypertensive individuals [30]. In comparison, we found that blood pressure was controlled in 14.9% hypertensive adults in Shandong Province [18]. Uncontrolled blood pressure may increase the risk of MAU progression. Recent hypertension management guideline have recommended that patients of any age with MAU initiate pharmacologic treatment to lower blood pressure [8]. Therefore, more aggressive preventive and/or treatment strategies are needed to control blood pressure in hypertensive patients with MAU.

MAU is the first sign of diabetic nephropathy. The percentage of our diabetic patients with MAU was much lower than the previously reported prevalence in China and other countries [31–33]. The prevalence of MAU was associated with disease duration [34]. Previous studies, however, usually included hospital-based patients or those with diabetes, with most of these subjects being patients with a history of diabetes. In contrast, most (70%) of the diabetic patients participated in our study were initially diagnosed in this survey. Furthermore, those previously diagnosed had a relative short disease duration (average 2.4 years).

We also found that MAU was associated with obesity and dyslipidemia [35, 36], with the prevalence of MAU strongly related to the number of established cardiovascular disease risk factors. The Metabolic Syndrome—a cluster of the risk factors including elevated blood pressure, fasting blood glucose, BMI and serum cholesterol—was shown to be a predictor of MAU progression, and eventually of overt proteinuira and chronic kidney disease [37, 38].

The utility of albuminuira screening in the general population is unclear [12, 14, 39–42]. Despite its predictive value for end-stage kidney disease, cardiovascular disease and death, several factors influence the potential implementation of MAU screening in clinical practice, including the prevalence of MAU in the target population, the type of test performed, the threshold for albuminuria and the cost of screening [39–42]. Results from the Third National Health and Nutrition Examination Survey finding in the United States estimated that screening of 11 (95% CI: 10–12) adults was required to identify one individual with MAU [14]. Our finding in adults from the Shandong Province suggested that 24 (95% CI: 20–30) adults would have to be screened to identify one with MAU. Studies are needed to determine the value of screening for MAU in the general Chinese population.

A major strength of our study was our use of the gold standard 24-h UAE to classify individuals with microalbuminuria, unlike previous studies in Chinese populations. Furthermore, this study included a representative sample of the general population, using rigorously standardized methods for data collection and strict quality control. However, our study has several limitations. First, we collected only one 24-h urine sample per participant, thus preventing a determination of day-to-day variability for each individual [43]. Second, the completeness of 24-h urine collection was assessed by measuring urinary volume and creatinine concentration. Without an objective measure such as p-aminobenzoic acid (PABA), it is difficult to assess the completeness of urine collection [44]. Third, serum creatinine was not measured in our study, therefore, we were unable to determine glomerular filtration rate or the prevalence of chronic kidney disease. Fourth, our study population included only adult residents of Shandong Province, thus limiting the applicability of our results to other populations. Finally, owing to the cross-sectional nature of this study, we were unable to quantify the associations betweenhypertension and diabetes and the progression of MAU. Follow-up studies are therefore recommended.

## Conclusions

This study showed that the prevalence of MAU in the general adult population of Shandong Province, China, is moderate. Rates of MAU were higher in subjects with than without hypertension and diabetes, with these two factors together associated with MAU. Screening of subjects with hypertension and/or diabetes for MAU may result in earlier diagnosis and treatment.

## Abbreviations

MAU: Microalbuminuria
UAE: Urinary albumin excretion
ACR: Albumin to creatinine ratio
UAC: Urinary albumin concentration
BMI: Body mass index
SBP: Systolic blood pressure
DBP: Diastolic blood pressure
FBG: Fasting blood glucose
OGTT: Oral glucose tolerance test
2hPBG: 2-h postload blood glucose
TC: Total cholesterol
HDL-C: High-density lipoproteins cholesterol
LDL-C: Low-density lipoproteins cholesterol
TG: Triglycerides
SD: Standard deviation
Q: Quartile
P: Percentile
CI: Confidence interval
OR: Odds ratio.
